# Impact of social and cultural factors on incidence, transmission and control of Coronavirus disease in Iran: a qualitative study

**DOI:** 10.1186/s12889-022-14805-2

**Published:** 2022-12-15

**Authors:** Saeed Falla-Aliabadi, Ahad Heydari, Farin Fatemi, Nooshin Yoshany, Mohammad Hasan Lotfi, Alireza Sarsangi, Fahad Hanna

**Affiliations:** 1 Department of Health in Emergencies and Disasters, School of Public Health, Shahid Sadoughi University of Medical Sciences, Yazd, Iran; 2 Accident Prevention and Crisis Research Center, Shahid Sadoughi University of Medical Sciences, Yazd, Iran; 3 Department of Health in Disaster and Emergencies, School of Medicine, Kurdistan University of Medical Sciences, Sanandaj, Iran; 4 Social Determinant of Health Research Center, Semnan University of Medical Sciences, Semnan, Iran; 5 Department of Health education and Promotion, Social Determinants of Health Research Center, School of Public Health, Shahid Sadoughi University of Medical Sciences, Yazd, Iran; 6 Departments of Biostatistics and Epidemiology, School of Public Health, Shahid Sadoughi University of Medical Sciences, Yazd, Iran; 7 GIS and Remote Sensing Department, University of Tehran, Tehran, Iran; 8 Program of Public Health, Torrens University Australia, Melbourne, VIC Australia; 9 Higher Education College, Chisholm Institute, Dandenong, VIC Australia

**Keywords:** Covid-19, Social dterminants, Cultural factors, Governance, Health policy

## Abstract

**Introduction:**

COVID-19 pandemic has had mixed reactions from nations, people and governments about ways to cope with, prevent and control the disease. The current study identifies social, cultural and policy factors affecting the incidence and control of Coronavirus disease in Iran.

**Methods:**

A qualitative study consists of content analysis as well as the views of 20 experienced and knowledgeable subjects specialized in social and cultural health management. The data were gathered using three semi-structured interviews and then continued by 17 semi-structured interviews. Data analysis was done using Graneheim approach. After each interview, the recorded audio files transcript and reviewed. Then codes extracted and divided to categories and sub-categories.

**Results:**

There are distinct social and cultural factors in coping with Coronavirus disease. These consisted of three categories of governance, individual and community related factors. A total of 17 subcategories and 215 primary codes that were extracted from the text of interviews as variables of the study and in relation to the research question. Ten subdomains of governance including vaccination, political issues, knowledge, support services, administrative services, transportation, health and treatment, culturalization, legislation and, managerial and financial policies impacted the spread and mitigation of the pandemic at various levels.

**Conclusion:**

The management of pandemics requires a comprehensive capacity for identifying and determining social and cultural criteria. A healthy partnership between governments and the community may be required to remove unnecessary obstacles that hinder public health attempt to alleviate the risk. The obtained criteria and indicators from this study may be utilized by policy makers in an attempt to strengthen protocols for mitigating pandemics. Further studies may be warranted to confirm these findings.

**Supplementary Information:**

The online version contains supplementary material available at 10.1186/s12889-022-14805-2.

## Introduction

Concurrent  with Coronavirus disease pandemic in the world, distinct factors based on the social and cultural characteristics of communities have undoubtedly impacted the rate of spread and the level of mortality of the disease in different parts of the world. In various geographical regions, people have displayed different behaviors in coping with the emergent diseases [1]. These behaviors are derived from social, economic and cultural processes and they are effective in the manner of reacting to that phenomenon and managing the consequences of that phenomenon, and, will pave the way for increasing and decreasing the vulnerability rate of communities to that phenomenon [2]. Factors such as education level, income level, security status, cultural status, the existent of indigenous and immigrant population can have an impact on increasing the vulnerability of community to emergent events [3, 4]. Being cognizant of cultural and social factors in different communities contribute to managers and organizational decision makers to take measures in line with reducing the possible harms based on available resources [5].

Considering the existent of various cultures and different social and economic conditions with adjacent countries, Iran has struggled with disease management and treatment of patients since the early months of Coronavirus disease. The official confirmation of the existent of positive test report form of Corona in Iran occurred in February 2020 leading the number of infections and deaths to culminate by March, June, November 2020, and at the months of April and August 2021 [6–9]. Given the mortality rate derived from the disease, various approaches were proposed by policy makers for disease management, that followed by a part of the community accompaniment and occasionally the existent of contradictory behaviors with the official policies of the government were seen by a part of the community [10]. These behaviors have been resulted from multifarious social, cultural and economic factors that it is required to be identified through distinct studies. By identifying these factors and examining their role in the incidence and control of Coronavirus disease, it can be taken action in line with applying management policies related to controlling the disease. These determinants can be compared with social and cultural factors in other parts of the world and can investigate the effect of cultural and social factors on disease management. Furthermore, examining the behaviors of community affected by social and cultural factors can be generalized to other emergent diseases that may transpire in the future.

Ample studies have been implemented on the role of social, economic and cultural factors in the incidence of infectious diseases in various parts of the world [11–16]. Concurrent with the coronavirus epidemic, considerable studies have been conducted in the domain of social issues given to the effect of this disease on population health. Other studies have examined the social vulnerability of communities resulted from the Coronavirus epidemic and presented the plan for removing the vulnerability [17–19]. Some of these essays used Social Vulnerability Index (SVI) data that encompasses 4 domains of socioeconomic status, household composition and disability, racial / ethnic minority and English language proficiency status, and housing and transportation and compared the vulnerability status of different places or groups [18, 20–22].

Some of the studies have investigated socio-cultural determinants related to Coronavirus via a systematic review. For instance, Chu et al. In a study have identified the social consequences of quarantine in communities during the infectious diseases pandemic period and presented strategies to reduce the negative social consequences of quarantine during the COVID-19 epidemic via systematic review [23]. In another study, the authors have examined the intervention rate of factors related to the social health in predicting epidemic consequences in models connected with COVID-19 [24]. Other researchers have examined the effects of social distance on the living conditions of various people as children, pregnant women, the elderly and patients during the corona epidemic period [25–31]. Moreover in some essays the effect of quarantine and distance on the incidence of social anomalies and behavioral problems has been stated [32–36]. In another essay, researchers have examined the social and economic factors of the COVID epidemic in 42 Asian countries empirically. In this study, the role of factors as age, status of background diseases, income, poverty, social capital, migration, social participation in the rate of getting infected and in the epidemic of the disease have been investigated [37]. On the other hand, the numbers of essays have been conducted in different countries of the world along with a qualitative approach in the domain of social conditions and its relationship with the issue of Coronavirus epidemics that examines the incidence of behaviors [31, 38–41].

Therefore, given to the importance of identifying socio-cultural factors affecting the incidence, transmission and management of Coronavirus disease in Iran, and because the culture, habits and beliefs of societies are different from each other. In this study, we tried to extract native factors that affecting the spread of Corona virus from the point of view of entomologists and experts of the society. It seems that qualitative study with content analysis method gives us better results than other methods. The purpose of the current study is to identify the social and cultural criteria and factors affecting the incidence and control of Covid-19 in Iran.

## Materials and methods

Since qualitative studies are very appropriate for identifying prominent variables related to a phenomenon, this study has been conducted qualitatively to identify the economic and social factors affecting the behavior of individuals during the Coronavirus pandemic period in Iran in 2021.

### Study population

In this study experts, managers and specialists who had knowledge, expertise and experience in multifarious domains, namely: health and treatment, epidemiology, sociology, political science, economics, cultural studies and religious experts from three regions of Iran from Kurdistan, Yazd and Tehran provinces were included for interview. The researchers contacted with the experts and explained the purpose of the study. If they were interested to participate in this research, they would invite for the interview. The semi-structured interviews were conducted purposefully in order to identify other experts and clear-sighted individuals, the snowball sampling method was also used for selecting participants. Entrance criteria for interviewees included possessing at least a master's degree in the relevant and above-mentioned domains, living in Iran, inclining to cooperate in the present study and encompassing an administrative background in the above-mentioned domains.

### Data collection method

In this study, after purposeful selection of population, face-to-face audio recording by MP3 Player was implemented through the interview guide. In total, 20 interviews were conducted. At the first, three semi-structured interviews were complemented due to specifying the pivots of the interview and completing the questions of interview guide besides the interviews continued along with 17 other interviews (Table 1). Data saturation obtained when no new themes or information emerged in subsequent interviews [42]. At the outset of the interview, the purpose of the study was explained to the populations. At the end of the interview, participants were requested to introduce other experts if they knew therefore the data can be validated by selecting clear-sighted individuals and spending enough time. Interviews were implemented between June and August 2021. The interviews prolonged between 20 and 50 min and averagely 30 min. Interviews were implemented by arrange a previous appointment and in a calm and quiet environment. To ensure the accuracy of the data and the interviewer's domination to a great extent as well, the audio files were listened at many times and if there were ambiguous topics or it was revealed the vagueness in formation of new questions, the interviewees would be contacted again. Eventually, the audio files were implemented in word software.

**Table 1:** Questions of interview guide used in the study

No	Questions
1	What is the effect of social and cultural factors on the spread, transmission and control of the Covid-19 disease?
2	In your opinion, what are the social and cultural factors affecting the spread, transmission and control of the Covid-19 disease?
3	To what extent can social and cultural factors play a role in the success or failure of corona control policies?
4	What solutions do you suggest to implement at the local and national level in order to prevent the incidence of the corona disease?

### Data analysis

Data collection and data analysis were implemented concurrently. Content analysis was conducted by Graneheim and Lundman method during 8 steps. Moreover the principles of qualitative content analysis of the recommended Graneheim were applied [43].

In this study, after conducting each interview, the text of the interviews was implemented at the first opportunity and the audio file of the interviews was typed. From the units of concepts and meaning, the units of the abridged meaning and then the primary codes were obtained. After that the codes based on a central concept were embedded within a subclass. Then, the sub-classes were revised at many times and compared with each other according to similarities and differences, and then the classes and the themes were existed, as there was the most homogeneity within the classes and the most heterogeneity between classes.

### Data credibility

In order to ensure the credibility of information, four criteria including credibility, dependability, transferability and conformability were determined [44].

### Ethical considerations

The time and location of the interviews were coordinated with the interviewees via phone calls. At the outset of the interview, participants were asked read and sign informed consent in relation to recording the interview and the researcher presented participant information sheet thereby ensuring that their recorded dialogue and conversations were confidential and that their first and last names to be anonymous and to be coded and the name of each individuals must not be known after implementing and converting into the text.

All experimental protocols in this study were approved by the Research Ethics Committee of Shahid Sadoughi University of Medical Sciences, Yazd, Iran (IR.SSU.REC.1400.052). Moreover, all methods were carried out in accordance with relevant guidelines and regulations.

## Results

The age of study population was between 34–65 years with an average age of 43 years. Three participants had MA degrees while 17 possessed Ph.D degrees. In accordance with the purpose of the study, 215 relevant codes were emitted from the content analysis of the questionnaires that in the subsequent step, these codes were divided into 3 main domains including governance factors, individual factors and the factors related to the community and were classified into 20 subdomains (Table 2).

**Table 2:** Demographic information of interviewees’ in the study

**Participants code**	**Age**	**Work experience**	**E**ducation	**Field of Study**	**Position**
1	35	7	Ph.D student	Health in disaster and emergencies	search and rescue officer
2	35	6	Master degree	Health education	Health center staff
3	40	17	Master degree	Nursing	Hospital supervisor
4	45	22	Ph.D student	Health education	Health center manager
5	39	15	Ph.D degree	Health in disaster and emergencies	Assistant professor
6	34	6	Ph.D degree	Health in disaster and emergencies	Assistant professor
7	34	11	Ph.D degree	Health in disaster and emergencies	Assistant professor
8	34	6	Ph.D degree	Nursing	Head of hospital
9	44	22	Master degree	Political science	Governor staff
10	46	26	Master degree	Physician	Head of hospital
11	45	21	Ph.D degree	Management	Governor's employee
12	46	23	Ph.D degree	Sociologist	Rescue officer
13	40	18	Ph.D degree	Surgery	Head of hospital
14	39	17	Ph.D degree	Environmental health	Health center staff
15	41	20	Ph.D degree	Psychologist	Assistant professor
16	39	19	Ph.D degree	Epidemiology	Disease control staff
17	34	5	Ph.D degree	Health education	Assistant professor
18	37	14	Ph.D degree	Environmental health	Health center head
19	65	35	Ph.D degree	Epidemiology	Retired
20	34	11	Ph.D degree	Statistics	Health center staff

### Governance factors

In this study, governance factors were identified as the most considerable and comprehensive component affecting the Coronavirus epidemic. Governance factors encompasses 10 subdomains of vaccination, political issues, support services, administrative services, transportation, health and treatment, culturalization, legislation, managerial and financial policies.

### Vaccination

Vaccination, as one of the vital strategies to reduce the spread of Coronavirus epidemic, has struggled with many challenges in Iran. Multifarious factors such as absence of public accessibility to vaccines, the loss of valid vaccines and the lack of public satisfaction to the vaccination have significantly hindered the public health efforts to mitigate the impact of the epidemic. To this point, participant number 14 commented as follow:

"All of the people had no access to the vaccine at the exact time, and at the very beginning the process of vaccination had started with the older age groups. These conditions have caused many people who did not reach the time for their vaccine turn to be frustrated, and occasionally because they were tired of following the protocol, they didn't incline to follow the limitations. The individuals whose relatives died of Coronavirus were depressed and some objected to these existent conditions." ( Participant 14, Environmental Health, age: 39).

### Political issues

Given the existent of political issues in Iran, and the sour international relations and challenges with other countries, as well as the existence of bank and logistic sanctions, the import of high quality and valid vaccines has been limited. Participant number 9 comment in this regard is noted below:

"For instance, our country cannot receive vaccines or health materials and products from developed countries because of international sanctions, and this has caused the process of vaccination and treatment to be difficult. On the other hand, the government officials promised that the government will produce Iranian vaccine that is not clear how effective it will be according to their statements as we are self-sufficient versus other countries that do not send vaccines to us. Some diplomats recommend holding political and religious demonstrations and other ceremonies as gatherings and elections." (participant 9, Education: Master in political science, age:44).

### Legislation

Ratification of legal restrictions is another obtained subdomain in this study. The results of this study demonstrate that factors such as entry of travelers from foreign country without testing and quarantining, the weak implementation the commute restrictions, non-obedience of certain groups from the enacted restrictions, absence of responsible organizations action toward health offenders and lack of staff due to controlling the Implementation of restrictions contributed to the increase of the spread of the pandemic.

In this regard, participants number 7 and 20 stated the following, respectively:

"Coronavirus disease is a disease that, given to the essence and nature of the disease, we should be able to prevent from spreading it correctly. We should block it. Blocking should be implemented in a way that martial law must be ordained. Commuting must be limited, in other words the governance must be powerful enough to convince the community that they must stay home. It must be closed everywhere for at least twenty days or one month; when it is blocked for a month or twenty days, the spread of the disease will automatically reduce, and if the governance does not possess this power, this can lead to spreading the disease to a great extent."( Participant 7, Ph.D in Health in disaster and emergencies, age: 34).

"The community is expected to follow the protocols, to the same extent, the government is expected to manage the disease by establishing coherent regulation and maintaining a coherent approach. If a single protocol and word is implemented equally to all of the individuals, the people will obey the regulations to a great extent." ( Participant 7, Ph.D in Statistics, age: 34).

### Knowledge

The results of this study demonstrated that in many situations, people may not follow health protocols due to loss of awareness about the nature of the disease and the ways of its transmission, or, in many cases people abstain from vaccination due to spreading propaganda and lack of adequate training, as per participant number 4 statement below:

"Another factor is related to the knowledge and getting aware of the disease. If we do not have sufficient knowledge and awareness and do not follow the principles of health, this will lead to the spread and transmission of the disease to a great extent, and this can be rooted in beliefs of people. We have knowledge and awareness in some cases but we do not believe, it means that we do not understand the dangers of the disease, in other words we do not know what is threatening us, and we do not feel the danger greatly." ( Participant 14, Environmental Health, age: 39).

### Administrative, support and transportation services

The findings of this study demonstrated that representing administrative services in a virtual way and the public and support services considered by the government for the people could encourage people to stay at home. This may reduce the volume of traffic and decrease the risk of encountering with the virus in the community. In this regard, participants number 2, 5 and 17 stated as follow, respectively:

"In many cases, people throng in banks or organizations and they confront with each other in indoor spaces. Unfortunately, electronic services are not presented to the people and they have to go to these places to meet their administrative and banking needs. On the other hand they have to travel from city to city, and when they travel, they may encounter with the people who are carrier of the disease in a taxi or bus." ( Participant 2, Master in Health education, age: 35).

"The expense of living in big cities is high. In this situation, the expenses of the conditions related to the Coronavirus disease prevention and treatment are imposed on people as well. Particularly in big cities, it is hard to say that people should not use the subway or bus. These transportation vehicles are also crowded and people do not obey the distance when they sit or stand beside each other." ( Participant 5, Ph.D in Health in disaster and emergencies, age: 39).

"Furthermore we should provide the minimum supplies and necessaries of the people. When we say stores should be closed, as some people do not possess good financial capability. At least affluent people or the government can meet their supplies and needs. Eventually, people need income. Their stores cannot be closed by us unless they do not have financial problems. If the government can contribute to them, fewer people will leave their homes." ( Participant 17, Ph.D in Health educatopn, age: 39).

### Health and treatment services

The preparedness of the health care system has a vital role in controlling Coronavirus disease. The health and treatment system must be able to prepare for identifying and screening the patients and this system must possess sufficient equipment, resources, personnel and hospital beds in the case of the outset of epidemic. In this regard, participant number 14 commented:

"The weakness of the health and treatment system can also be effective in the expansion of the disease. If we do not have diagnostic test and do not be able to screen it will cause the expansion of the disease to a great extent and if our health and treatment system cannot be able to treat accurately, it can pave the way for disease epidemic. Obeying from the health protocols in places as restaurant or shop can be effective in incidence of the disease." ( Participant 14, Environmental Health, age: 39).

### Culturalization

Culture of the community is one of the vital factors in controlling or expanding diseases as Covid-19. Despite the efforts, it has not been possible for the Iranian government to develop a culture of Covid-19 to a great extent. In many cases, the implementation of the established restrictions and laws was hindered by this issue. In this regard, participant number 5 commented:

"The first measure that should be taken in the country via schools and the media is to build a culture in the domain of personal care so that people can notify of the prevention ways. At the very beginning there were involvement of news, media, radio and social media and systemic recommendations were given to the people for disease prevention. It is propagated contradictory statements about the nature of the disease and how to cope with it, especially in social media, and some people believe them though." ( Participant 5, Ph.D in Health in disaster and emergencies, age: 39).

### Managerial and financial policies

Financial and economic policies of the government are effective in controlling the disease to a great extent. Financial contribution of the government as bank cooperation, deferred debt, strengthening charitable organizations financially and the existence of support organizations as the Relief and Welfare Committee to help the families under coverage of the insurance can effect on preventing the expansion of the disease. In this regard, participant number 3 stated:

"Even for the people who live in slums, the existence of health packages as masks and sanitizers can be very effective. There are people who live in difficulty and want to obey the regulations but they cannot follow the route. Financial contributions as providing loan and livelihood contributions and it must be taken action not just words”. ( Participant 3, nursing, age: 40).

### Individual factors

The domain of individual factors was broached as an effective element in relation to Coronavirus epidemic by the interviewers in this study. Individual factors were stratified into 7 subdomains after categorizing and combining common codes.

### Understanding the risk

Understanding the risk of people in community and being aware of the consequences of high-risk behaviors is one of the most vital factors affecting the disease epidemic. On the other hand, the absence of knowledge and awareness of people about the disease, its consequences and control strategies can reduce the understanding the risk of people. In this regard participants Number 1 and 19 commented, respectively:

"Some people as youth or athletes possess the exaggerated courage or fearless as if I am in good condition and I will never get sick and in case of getting infected I will overcome the disease. This wishful thinking and misconception have embedded in his mind and have paved the way for not wearing mask." ( Participant 1, Ph.D student of Health in disaster and emergencies, age: 35).

"Some people soliloquized that I should call on my friend and relatives and greet them but he did not know that if he has been the carrier of the disease, he would have entangled some other people and if God forbid, the same friends or relatives to be died of the disease, the one will not forgive himself." ( Participant 19, Ph.D in statistics, age: 65).

### Personality factors

The behavior of the people is derived from their personality. People may be dissociable or be possessed high social relationships. A group of people may be parsimonious enough to not buy masks and prevention instruments related to Covid-19. Some people are depressed and tired of following the health protocols and restrictions. These personality features are effective on the behaviors of the people towards prevention from Covid-19. In this regard participant number 6 commented:

"Even some people said apparently that we wear a mask; but it does not go with us and we like to refuse wearing masks. When we got infected to Coronavirus, it is not a problem as we will be treated. Some individuals are tired of this situation and say that I how late I should obey. Maybe this disease may be existed forever. Of course some individuals got along with this condition and accept the Corona protocols as a part of their life." ( Participant 6, Ph.D in Health in disaster and emergencies, age: 34).

### life style

Another factor affecting on high-risk behaviors during the corona epidemic is lifestyle. Some people live in thickly populated families and communities. Some people smoke, consume alcohol or use drugs, or some people may be weakened physically due to poor condition of nutrition and also their body may be more vulnerable. In this regard participant number 18 commented:

"Some people look the disease more seriously and maybe obey the protocol to a great extent because of their vulnerabilities, as poor nutrition or living at home with the elderly." ( Participant 18, Ph.D in Environmental Health, age: 37).

### Health condition

The results of this study demonstrate that health condition of people can affect their behavior before Coronavirus disease. People with rare diseases and also the people who are exposed to the risk of health follow health protocols more than the people who are in good health condition. In contrast, the youth and athletes who are in a healthy physical condition show high-risk behaviors before the disease. The participant number 12 commented:

"Families with people who are infected with chronic disease as diabetes, cancer and background diseases, family members try to obey the health protocols to a great extent and also it is seen less commuting in these families." ( Participant 12, Ph.D in Sociology, age: 40).

### Factors related to the community

In this study, a group of emitted codes regarding the factors affecting the epidemic of Covid-19 in the community were classified into subdomains of social and cultural factors and were embedded in the domain of community-related factors.

### Social factors

Social behaviors as companionships, parties, traveling and visiting are considered as social behaviors related to community norms. Various ethnicities have their own social rituals and behaviors. Moreover, traditional communities in smaller cities rely on their mores to a great extent and do not incline to stop these mores. In this regard participant number 8 and 13 commented as follows, respectively:

"We are a traditional community. We do not quit a series of habits, behaviors and ceremonies, including weddings and funeral ceremonies easily. These situations can pave the way for a more expansion of the disease." ( Participant 8, Ph.D in Nursing, age: 36).

"At the beginning of the disease epidemic, many people quitted important and traditional ceremonies as visiting in Nowruz holidays, as Corona period had been lasted and perhaps the situation had been normalized for the people, this ceremony was held again, of course this ceremony was hardly implemented in the next year." ( Participant 13, Ph.D in Surgery, age: 40).

### Cultural factors

The culture of communities is one of the most vital factors that can affect the behavior of people. In general, some communities are circumspect and in some other communities, due to the existence of prevailed and old culture of high risk-taking, the high-risk behaviors are common among individuals. On the other hand, changing the culture in some communities is very tough and time consuming, for instance, canceling and avoiding parties and some traditional ceremonies during the disease epidemic. In this regard participant number 11 stated:

"It is probable that some ethnicities and cities may have their own series of regulations. In this condition, it is not important how hard you will attempt as the restrictions are before their culture, you cannot really hope to see following them therefore in everywhere we must know their own cultures and impose restrictions on them." ( Participant 11, Ph.D in management, age: 45).

## Discussion

According to the results of this study, the obtained components along with positive and negative effects on the incidence, transmission and control of coronavirus are embedded in three main categories of governance, individual and social components. Policy-making, making legislation and community awareness about corona disease are the issues that are considered as the most vital components of governance that are undertaken by the government of Iran and the Ministry of Health, in particular. Implementing inappropriate policies or making decisions in inappropriate time by the government may have led to increased rate of mortality and lack of trust of the people towards their government and the privation of resources. For instance, delays in the arrival of a valid vaccine led to relatively high rate of mortality (700 people in August2021) [9]. A study in India on rate of mortality in first and second peak demonstrated that the rate of mortality in the second peak was lower than the first peak as part of population over 65 years of age being vaccinated in the second peak compared to the first peak of the disease [45]. At the same time, contradictions about the arrival of foreign vaccines revealed, and some other broached ineffectiveness of the vaccination or its negative effect on community health as well thereby negatively influencing the acceptance of members of the community to vaccinate. This issue was quite prevalent in some Iranian regions along with the people of Japan and the United States has been discussed in a study between 17 countries of the world [46]. Additionally, considering the lack of sufficient vaccines in Iran, the process of vaccination has been categorized and it takes a long time for each individual in the community to be vaccinated, which has resulted into the slowing control of Coronavirus.

Another issue is legislation of governance aiming to manage and reduce the rate of Corona disease. Lack of attention paying and adherence to the setting of restrictions and regulations by non-experts may pave the way for ineffectiveness of such legislation. Following the high proportion of people who got infected in Iran, lengthy periods of restriction were announced in various cities around the country. These restrictions were accompanied by closing various places and prohibiting public gatherings, however, the absence of serious mitigation, planning and compensation resulted into abundant economic problems for the public. Simultaneously, the government had to provide financial subsistence and support for people to stay at home thereby reducing the potential violation of restrictions due to economic pressures. On the other hand, providing financial subsistence support packages could have a significant effect on reducing public commuting but finally, poor support policies and social factors by the government caused people to defy the restrictions, which ultimately contributed to increase rate of infection and mortality. Further, social factors caused psychological problem, depression and violence among people [36]. These findings are consistent with the results of a study conducted in Santiago, Chile in 2021. The mentioned study demonstrated that there is a strong and significant relationship between social-economic status and the effects of Covid-19 and capacity of community health [47]. Our study also found that the deprivation of various services to violators of health protocols and effective monitoring of operation in community may pave the way for reducing the epidemic of the disease, transmission and ultimately the rate of mortality. Moreover another predicament of the legislation is that there is no coherent management for implementation of regulations, and the behavior of the managers indicates dual personality/views toward implementation of regulation. This finding is consistent with a study in Sweden by Modig et al. (2021) where the government restrictions being “recommended” rather than strictly implemented may have contributed to higher rate of mortality due to Covid-19 infection [48]. On the contrary, in various other countries, strict regulations were imposed on the public which resulted into a more efficient way for controlling the spread of the disease [48, 49]. For instance, the appropriate enforcement and control of the social distance policy in some countries, such as Italy, has successfully reduced the rate of mortality in the second peak of Covid-19 disease in the country [49]. Distribution of livelihood packages, loan payments, supporting of low-income families who lost livelihoods, supporting and withholding loan repayment, saving individuals and families from business bankruptcy, price reduction of essential materials such as medicine and main nutrients should be taken on board to encourage the public to follow the pandemic rules. A county-level analysis in the United States demonstrated that regions with lower economic status had been faced with higher rate of mortality in the Coronavirus pandemic [50]. Furthermore, a more extreme example of poverty in Peru was considered as one of the most influential factors for the excessive rate of mortality due to Covid-19 disease [51]. The issue of lack of support from the government of Iran at the economic level, particularly not considering those with poor economic conditions, has also been discussed in another study [52].

Informing the community of the prevention from the pandemic, treatment, and consequences of Covid-19 requires the all-out participation and involvement of health care providers, non-governmental organizations, donors, celebrities, and religious and faith leaders [53]. Considering the fact that medical providers and frontline healthcare workers would struggle with a long-term pandemic, it is imperative to provide sufficient economic, occupational and psychological support to this cadre. In many cases the health and treatment system in Iran has encountered with the lack of medicine, hospital beds, oxygen and health staff including physician and nurses in management and controlling of the disease at the beginning of the expansion of Covid-19 [54]. High cost of treatment and prevention have been another significant problem. The relative lack of familiarity with the virus and the use of different treatment protocols, with some being ineffective, has led to community pessimism about the treatment cadre, which occasionally caused people to avoid hospitalization leading to condition being exacerbated. A study conducted Iranian cities demonstrated that lack of cure along with other rumors may have paved the way for fear of this epidemic among the public [55]. Assuring the public about the effectiveness of medical treatment in early and timely hospitalization may require a health partnership between governments and the community as a whole.

Another important factor in this study that highlights the role of community in controlling the disease was the individual, cultural and social characteristics. The culture of the people in Iran is such a way that it is mandatory to companion (visiting and meeting the elders of the family) or to participate in weddings or funeral ceremonies that these behaviors increase the incidence and expansion of this disease in the community. Many of the emerging data on COVID-19 death seemed to be pointing to the fact that older and unhealthy people were more likely to die from it. This was specifically highlighted in a study conducted in Ardebil (Iran), where one of the most predominant mortality risk factors age [56]. A proportion of the public ignored the disease by violating the regulations and/or by claiming to be protected due to being young or athletic. The results of a study in the Ohio and Florida states of US between March 15 and December 5, 2020 demonstrated that higher than expected rate of mortality have occurred at the age group of 20–49 years [57]. Fatalism, arrogance, insistence, getting susceptible to depression, escaping from loneliness and getting tired of obeying protocols and constant use of masks are considered as other individual and behavioral factors that led to disobeying or neglecting health protocols. Profession type of individuals was also a significant factor in controlling and preventing Coronavirus in community. Findings from the same (above) US study demonstrated that compared with whites, black people, who are placed in a lower economic status and whose professions could not be telecommuted/virtual, exhibited higher mortality rate from the virus [57]. The number of clients, size of indoor or outdoor spaces, population density and the degree of obeying the protocols in the workplace can affect the spread and transmission of the disease. At the beginning of the Corona epidemic in Iran, medical staff, taxi drivers, and bank employees had the highest rate of infection and mortality due to COVID-19, however, the rate of death decreased in these groups concurrent with prioritizing vaccination for these individuals [58].One of the limitations of this study is that being qualitative is more based on individual judgment and opinion and is less likely to be representative of the general Iranian population.. The information derived from qualitative research can also be highly subjective. However, we have used all efforts to ensure accuracy of data collected from individuals who simply expressed their views about the pandemic. A more quantitative approach coupled with larger sample size and longitudinal design may provide more accurate and reliable data. Furthermore, these findings are relevant to Iranian communities may not necessarily apply to other countries and communities. However, poverty, cultural practices, behavior and other individual characteristics can be common human features, particularly in the developing world.

## Conclusion

Managing the COVID pandemic requires a comprehensive knowledge and understanding of social and cultural determinants within each community to facilitate more comprehensive protocols and approaches to mitigate the spread. It is mandatory to provide health, economic and operational infrastructure and support to further enhance the management and control of the pandemic. Moreover, the results of this study demonstrate that in order to improve the effectiveness of disease prevention, managing and control, a more comprehensive cooperation and coordination between all departmental and intradepartmental organizations as well as various institutions of the country, together with a healthy partnership with the community is required. Therefore, it is highly recommended that trainings, public and specific regulations, programs and the needed resources must be enforced and compiled for preparing and responding to epidemics relevant to the economic and cultural status of the community. The findings of this study can be utilized by policy makers in the domain of cultural, social and health issues in relation with the management of the Covid-19 pandemic and any future pandemic. Further studies mixed with quantitative data may be required to increase the credibility and generalizability of the determinants of Covid-19 spread and control.

## Supplementary Information

**Additional file 1.** **Supplementary Table 1. **Domains, subdomains and emitted codes regarding social and cultural factors affecting the Covid-19 pandemic.

## Data Availability

All data extracted from this qualitative study is available upon request. The majority of the raw data is in Persian, however, a translated version will be available upon request. The point of contact for this would be 1st author Saeed Falla-Aliabadi, Shahid Sadoughi University of Medical Sciences.
