# Supplementary material for: Impact of social and cultural factors on incidence, transmission and control of Coronavirus disease in Iran: a qualitative study
Source: BMC Public Health. 2022 Dec 15;22:2352. doi: 10.1186/s12889-022-14805-2 (PMC9753076; doi:10.1186/s12889-022-14805-2)
Supplement: Supplementary file 1 — Additional file 1. Supplementary Table 1. Domains, subdomains and emitted codes regarding social and cultural factors affecting the Covid-19 pandemic. [file 12889_2022_14805_MOESM1_ESM.docx]

**Appendix 1**

**Supplementary Table 1: Domains, subdomains and emitted codes regarding social and cultural factors affecting on the Covid-19 pandemic**

| **Domains** | **subdomains** | **impact** | **codes** |
| --- | --- | --- | --- |
| **Governance** | vaccination | Increasing | Lack of public access to vaccination, lack of assurance of people toward vaccines, creating black market of vaccines, stratified vaccines, absence of public cooperation with vaccination cadre, not obeying the announced turns of vaccination by the people, Lack of public assurance to domestic vaccination, absence of some people and executors assurance to foreign vaccination |
|  |  | Decreasing | Lack of people insistence to provide vaccine, enforcing encouragement policies for vaccinating, deprive of social services for the people who had not been vaccinated |
|  | Political | Increasing | The existence of economic and political sanctions against Iran in different domains as medicines and currency, vaccines arrival restrictions due to lack of some Iranian executors assurance to foreign vaccines, facilitating the holding of religious gathering, holding political gatherings, the arrival of Afghan refugees from the direction of Afghanistan border due to political problems in Afghanistan |
|  |  | Decreasing | Obeying the protocols related to the Coronavirus by religious leaders, lack of march holding or Friday prayers |
|  | Legislation | Increasing | Lack of enforcing restrictions in borders, arrival of travelers of foreign country without testing and quarantine, absence of enforcing legal restrictions based on evidence and expansion of the disease, lack of willingness of some people to obey the restrictions due to daily needs or economic issues, indecisiveness of organizations in imposing restrictions against health violators and offenders of imposed restrictions, lack of personnel for monitoring the restriction enforcement. |
|  |  | Decreasing | Closing of administrative, commercial, sports and educational places, prohibition of holding wedding ceremonies, restriction of attendance in funeral ceremony, changing or reducing the working times of organizations, clear and timely informing of restrictions, enforcing the appropriate social distance in community |

| **Domains** | **subdomains** | **impact** | **codes** |
| --- | --- | --- | --- |
| **Governance** | educational | Increasing | Unscientific statements of traditional medicine experts, lack of adequate information about the effectiveness of some methods related to the treatment or prevention from the disease |
|  |  | Decreasing | Creating awareness in the community via giving information about the disease and disease prevention methods through the media, organizations, mosques, health experts and health house, training about how to use masks and other personal protection equipment, training people how to cope with Coronavirus, informing the people of rumors, giving information about the effectiveness of the vaccine |
|  | Support Services | Decreasing | Distribution of health packages among the people by charitable organizations and donors, providing disinfection equipment in organizations, providing free masks for clients in organizations, providing masks in gatherings, preparing and producing high quality masks by home-based businesses, providing high quality masks, inexpensive and comfortable, providing household provisions for the families who caught the Corona disease, providing livelihood assistance packages by non-governmental organizations and donors for low-income families or for the people who do not receive income |
|  | Administrative services | Increasing | Absence of organization cooperation with people, delays in providing administrative services, absence of organization employee to a great extent, the need for physical presence of people in organizations for receiving the needed services, lack of implementing electronic services, lack of infrastructure in electronic administrative services, the existent large number of people for receiving administrative services, lack of scheduling system in administrative services |
|  |  | Decreasing | Providing distance bank services, increasing the maximum of distance bank services, decreasing distance education services |
|  | Transportation | Increasing | Failure to increase the number of buses and subways given to the population, increased cost of providing transportation services, not obeying the approved restriction on the number of passengers attendance in trains and intercity buses and planes, high population density in public transportation vehicles |
|  |  | Decreasing | Existent of traffic control plan |

| **Domains** | **subdomains** | **impact** | **codes** |
| --- | --- | --- | --- |
| **Governance** | Health and treatment | Increasing | Lack of timely attendance of people to hospitals and health and treatment centers, constant changing of treatment protocols, burning out of medical personnel, lack of presence of people to disease diagnostic laboratories |
|  |  | Decreasing | Participation of donors and non-governmental organizations in the construction of field hospitals and purchasing of medical equipment |
|  | culturalization | Increasing |  |
|  |  | Decreasing | Obeying the principles of quarantine and staying at home, utilizing social media, distance education, promoting the prevention culture, informing people of health violators and offenders of restrictions |
|  | Managerial | Increasing | Not keeping the promise of government for supporting the people who harmed and exposed to the risk of Coronavirus, hesitating in the enforcement of health protocols and imposed restrictions, indifference of officials toward problems of people during the epidemic of the disease, loss of attention to the disease prevention |
|  |  | Decreasing | Drawing attention of people toward governance in community, benchmarking from successful countries in controlling the disease, contingent decision making at different timescales of the disease epidemic in community |
|  | Financial Policies | Increasing | Lack of bank cooperation in domain of check collection and facilitating in repayment of the loans, bankruptcy of professions, reduction of government revenues, loss of strengthening charitable and aid organizations as the Relief committee and Welfare organization in the direction of emergency aid for the families are the coverage of the insurance |
|  |  | Decreasing | Confirming the financial support policies for people exposed to the coronavirus, allocating interest-free or low-interest bank loans, amnesty for financial and fiscal crimes |

| **Domains** | **subdomains** | **impact** | **codes** | |
| --- | --- | --- | --- | --- |
| **Individual** | Understanding the danger | Increasing | Relying upon being an athlete, claiming to be healthy, ignoring the disease, unrealistic relying upon the vaccine, prioritizing the primary and basic necessities of life, being indifference, normalization, expressing mendacious ignorance, showing reluctant to believe in the disease, possessing low hope to the life and danger reception, not believing in the existence of the disease and its consequences, attending in crowded places and medical centers, depending on education, being obstinate to obey the health protocols, getting tired of obeying the protocols and staying at home and not attending in ceremonies, intolerance of children to stay at home, normalizing the situation, being interested in attending ingathering, travelling to the prohibited or high-risk places in terms of the expansion of the disease, violating the protocols covertly, not accepting the recommendations, using the public transportation, imitating officials, visiting patients | |
|  |  | Decreasing | Obeying the principles of health and quarantine after getting infected, depending on education, timely replacing masks, recommending and notifying others regarding the obeying the health protocols and restrictions, imitating officials | |
|  | Personality factors | Increasing | Braveness, false courageousness, pride of youth, being stingy, bashfulness, altruism, fatalism, depression, people's indifference to obtaining knowledge about the disease, escaping from the loneliness | |
|  |  | Decreasing | Being law-abiding, accountability | |
|  | Professional and economic factors | Increasing | Not obeying social distance in the workplace, large number of clients, large crowd of staffs , existence of small space of workplace, closing of the business, delay in payment of the salary, unemployment, service suspension, low income, type of profession, preference of financial conditions over health , violating the regulation due to economic conditions, not being able to buy masks and other disease prevention equipment, income decrease, creditor pressure, check maturity, high expense of hospitalization, expense of using a personal car, high expenses of living, class distinctions, low area of the house, high expense of receiving services to the patients with Coronavirus disease at house, increased expenses of main material as medicine and basic foodstuffs, living in slums, not possessing personal house, living multi-family in one house. | |
|  |  | Decreasing | The type of profession, the vastness of workplace, existence of equipment and prevention facilities in workplace, obeying the health protocols by individuals in workplace, telecommuting, downsizing, financial contribution to each other, existence of many rooms in the house, possessing the personal car | |
| **Individual** | Life Style | Increasing | Drug use, not having sufficient time for following the news related to the disease, participation in parties, attendance in religious ceremonies |  |
|  |  | Decreasing | Appropriate nutrient, being athlete, following the health behaviors because of personal health |  |
|  | Health Status | Increasing | The weakness of immune system, possessing background disease |  |
|  |  | Decreasing | Possessing physical and mental health, accessing to physician, possessing medical case |  |

| **Domains** | **Subdomains** | **Impact** | **Codes** |
| --- | --- | --- | --- |
| **The factors related to the community** | Social factors | Increasing | Imitating the celebrities, imitating the adults, sending children to relatives' house, sending children to kindergarten, employed parents, imitating the educated people, not obeying the health protocols by family members and relatives, lack of respect for the elderly and visiting them, the large population of the family, the need for companionship, decreased social vitality, getting infected of family members to the disease, low awareness and literacy in part of the community, intolerance of community, community burning out, lack of community trust, large population of relatives, intimacy of relatives, holding family parties , immigrant community, tourist-friendly of the region, not quitting the usual social habits in the time before Corona, the existent of a traditional community, insisting on holding ceremonies, the need for receiving essential services, lack of access to media, medical tourism |
|  |  | Decreasing | Imitating the medical forces, imitating the celebrities, imitating the adults, imitating the educated people, obeying the health protocols in public places, the existence of high degree of awareness and literacy in part of the community, possessing a spirit of helping each other |
|  | Cultural factors | Increasing | Being multi-cultural, multi-ethnic, superstitions, being indifference to each other's health, shaking hands, kissing each other, attending in religious gatherings, insisting on implementing traditions as observation of relationship, visiting the patient, etc., holding different cultural and traditional ceremonies |
|  |  | Decreasing | Being law-abiding, cultural level, interdicting different people, high social consciousness, existent of religious beliefs |
